# Clinical landscape of macrophage-reprogramming cancer immunotherapies

**DOI:** 10.1038/s41416-024-02715-6

**Published:** 2024-06-03

**Authors:** Jenna H. Rannikko, Maija Hollmén

**Affiliations:** 1 MediCity Research Laboratory and InFLAMES Flagship, University of Turku, Turku, Finland; 2 Turku Doctoral Program of Molecular Medicine, University of Turku, Turku, Finland; 3 Faron Pharmaceuticals Ltd, Turku, Finland

**Keywords:** Innate immunity, Tumour immunology

## Abstract

Tumour-associated macrophages (TAMs) sustain a tumour-supporting and immunosuppressive milieu and therefore aggravate cancer prognosis. To modify TAM behaviour and unlock their anti-tumoural potential, novel TAM-reprogramming immunotherapies are being developed at an accelerating rate. At the same time, scientific discoveries have highlighted more sophisticated TAM phenotypes with complex biological functions and contradictory prognostic associations. To understand the evolving clinical landscape, we reviewed current and past clinically evaluated TAM-reprogramming cancer therapeutics and summarised almost 200 TAM-reprogramming agents investigated in more than 700 clinical trials. Observable overall trends include a high frequency of overlapping strategies against the same therapeutic targets, development of more complex strategies to improve previously ineffective approaches and reliance on combinatory strategies for efficacy. However, strong anti-tumour efficacy is uncommon, which encourages re-directing efforts on identifying biomarkers for eligible patient populations and comparing similar treatments earlier. Future endeavours will benefit from considering the shortcomings of past treatment strategies and accommodating the emerging complexity of TAM biology.

## Introduction

Tumour-associated macrophages (TAMs) make an alluring cancer immunotherapy target. For one, their abundance in tumours allows TAMs to greatly influence the interplay between cancer cells and the surrounding tumour microenvironment (TME), resulting in tumour-promoting milieu essential for cancer progression [1–5]. Furthermore, great expectations have been placed on targeting TAMs, as they were long thought to exist in and switch between two states, tumour-promoting immunosuppressive (M2-like) macrophages and anti-tumoural pro-inflammatory (M1-like) macrophages [6, 7]. Nowadays, it is known that the macrophage phenotype is more complex than the state between M1 and M2 macrophages, with TAMs displaying features of both [8–10]. Macrophages are shaped by their tissue environment, and while this regulates their functional responses, they also readily adapt to changes in environmental cues, which renders them susceptible to therapeutic manipulation [11–14].

Several approaches have been taken to unleash the therapeutic potential of macrophages, including TAM depletion strategies, preventing TAMs from entering tumours and reprogramming TAM phenotypes or functions from tumour-promoting to anti-tumoural [4, 15]. However, depleting TAMs with CSF1/CSF1R blockers has limited monotherapy efficacy, except in diffuse-type giant cell tumours that overexpress *CSF1* [16, 17], because the benefits from TAM depletion are counteracted by enhanced recruitment of granulocytic myeloid-derived suppressor cells (MDSCs) [16]. Similarly, inhibiting monocyte recruitment by blocking the CCL2–CCR2 axis induces compensatory recruitment of tumour-associated neutrophils [18], and CCL2 inhibition has also been associated with major increases in CCL2 levels and enhanced metastasis after therapy discontinuation [19, 20]. Simultaneous blockade of multiple complementary or compensatory chemotactic pathways is therefore investigated to counteract compensatory myeloid chemotaxis [18, 21]. Rather than equally targeting all macrophage subsets, TAM reprogramming aims to spare and support anti-tumour TAMs. This may alleviate potential side effects and promote stronger tumour rejection [22, 23].

In this review article, we discuss the potential of TAMs as cancer therapy targets, summarise past and present clinical development of TAM-reprogramming therapeutics and identify challenges and opportunities for developing macrophage-based therapeutics to reach their full potential in cancer treatment.

## TAMs as cancer therapy targets

A plethora of research has highlighted the various ways in which TAMs promote cancer development and progression, providing ample therapeutic opportunities. In short, TAMs act in every stage of the metastatic cascade by promoting primary tumour growth [24], angiogenesis [25], immune evasion [26], invasion [15, 27, 28] and metastatic spread [15, 29–31]. These TAM functions develop gradually when TAMs co-evolve with cancer and are based on homoeostasis-promoting functions of healthy macrophages [4]. Further emphasising their therapeutic potential, TAMs promote cancer immunotherapy resistance by limiting T-cell entry into tumours and suppressing anti-tumour T-cell activation [26, 32, 33]. However, TAMs can fight cancer by phagocytosis [34], killing with reactive radicals [35, 36] and activating anti-tumoural immunity via antigen presentation and cytokine secretion [37]. The long co-evolution with cancer cells ultimately dampens these anti-tumoural TAM properties [4], unless prevented with therapeutics.

Considering the tumour-promoting roles of TAMs, it is no surprise that a high abundance of TAMs is associated with poor prognosis in most solid cancers [38]. Colorectal and prostate cancer are exceptions to this, but when immunosuppressive M2 marker-expressing TAMs are quantified instead, a negative prognosis is evident across cancer types [38]. A high abundance of immunosuppressive TAMs is characteristic of so-called non-inflamed tumours that lack T cells and are often resistant to immune checkpoint inhibitor (ICI) therapy, further contributing to poorer prognosis [39]. However, these overall trends give a simplistic view, as the intratumoural localisation of TAMs and selected treatment regimen also affect their prognostic value. For instance, tumour islet TAMs are often associated with a more favourable prognosis than stromal TAMs [40–42], whereas the negative prognostic value of TAMs in pancreatic cancer is counteracted by adjuvant chemotherapy [43] and the positive prognostic value of TAMs in colorectal cancer is observed in 5-fluorouracil-treated patients [44]. Although these associations are most commonly based on CD68, a marker not entirely macrophage-specific, they show different functionalities in existing TAM populations and should be considered when targeting TAMs in different cancer types.

A further layer of complexity in TAM biology stems from their diversity. TAM phenotypes and functions are affected by both macrophage ontogeny and the local tissue microenvironment [10]. While most macrophages in many healthy tissues originate from embryonic precursors, tumours resembling inflamed tissues recruit monocytes that differentiate into macrophages, making TAMs a mixture of embryonic and monocyte-derived populations [45–49]. Once monocytes enter the cancer tissue, their phenotype is modified by the cancer type, affected organ and intratumoural localisation, as these determine the characteristics of local tissue niches. Factors such as neighbouring cells, matrix composition, pH and cytokine environment will then further fine-tune TAM phenotypes and functions [10, 26]. Furthermore, monocyte differentiation into macrophages is altered in cancer, resulting in additional accumulation of immature phenotypes, such as MDSCs [50].

Investigating TAM phenotypes in different tumour areas and cancer types is possible using single-cell and spatial analysis technologies. For instance, Ma et al. used existing datasets of single-cell RNA-sequenced TAMs and identified seven TAM phenotypes common to multiple cancers [10]. Interestingly, these identified TAM phenotypes have both pro- and anti-tumoural properties within the same subset, such as interferon (IFN)-primed TAMs that express IFN-regulated mediators contributing to both T-cell activation and exhaustion, and regulatory TAMs that resemble immunosuppressive M2-like macrophages with their PD-L1, IL-10 and MRC1 expression but also express co-stimulatory and major histocompatibility complex (MHC) molecules [10]. As expected, these TAM phenotypes strongly connect TAM functions with their localisation within the tumour. For instance, pro-angiogenic TAMs reside in hypoxic tumour areas, where they promote metastatic spread and angiogenesis [10], whereas immunosuppressive lipid-associated TAMs are located in areas of invasion, where they suppress T-cell responses [10, 51], and IFN-primed TAMs co-localise with CXCL13-expressing T cells to regulate their responses [10].

Clearly, these different and opposing functions of TAM subsets require tailored therapeutic targeting. It is yet to be determined which of these TAM subsets are susceptible to functional reprogramming and which are the most crucial to be reprogrammed. If these TAM subsets possess both anti- and pro-tumoural capacities, successful manipulation would promote their anti-tumoural functions without activating the pro-tumoural ones. Of note, depending on cancer type, the markers for these subsets may differ, and some of the widely investigated TAM molecules are associated with several subsets, such as TREM2 [10]. Nevertheless, uncovering TAM functions at the subset level will provide opportunities for developing even more sophisticated TAM-reprogramming therapeutic approaches.

## Clinical landscape analysis

To obtain a comprehensive understanding of the clinical landscape of TAM-reprogramming strategies, we gathered data from past and present clinical trials investigating TAM-reprogramming agents in cancer. For this, we first searched PubMed for potential TAM targets using the following search conditions: macrophage and (cancer or neoplasm or tumour or tumor or malignancies) and (phase or clinical or trial or target), where ‘macrophage’ and the cancer-related term had to be in the title or abstract. The obtained 17,457 unique articles published before 1.2.2024 were screened by Rayyan [52] using the following exclusion criteria: (1) the paper does not discuss or identify therapeutic targets, specifically for cancer and in macrophages; (2) the therapeutic strategy primarily depletes TAMs or inhibits their recruitment and (3) the therapeutic is re-purposed. Screening was performed mostly manually, with Rayyan-created ratings used to exclude the lowest matching articles only after screening one-third of all articles and confirming the irrelevance of the lowliest rated articles. The identified TAM targets and therapeutics from the obtained 550 review and research articles or the word ‘macrophage’ were then used as the search condition in clinicaltrials.gov to find clinically investigated TAM-reprogramming therapeutics (condition/disease = ‘Cancer’).

During the search, we excluded withdrawn trials and therapeutics with first trial start date before 1.1.2000, and expanded our original TAM target list with additional TAM-targeting therapeutics from the company pipelines encountered. In this way, 194 clinically evaluated TAM-reprogramming therapeutics were gathered with information on the clinical phase, number of trials, combinatory regimens, first clinical trial date, investigated cancer types and actual number of treated patients. Clinical development was classified as discontinued if the therapeutic had been removed from the company’s pipeline without information on selling the asset, most recent trials had been terminated, or trials had remained inactive for several years without new trials being launched. Additional information was collected from publicly available sources, including published conference abstracts, press releases, web-based company pipelines and quarterly reports. While we call these therapeutics ‘TAM reprogramming’ for clarity, many of them are not macrophage-specific and may have other functions, as described in the discussion of individual targets. We also acknowledge the presence of other crucial pathways controlling TAM phenotypes not discussed in this review. For instance, cytokines, prostaglandins and other inflammatory mediators have additional broad effects on various immune and non-immune cell types and represent potential strategies for modulating cancer-associated inflammation rather than specifically controlling TAMs.

## Past and present clinical trials

**Fig. 1:**
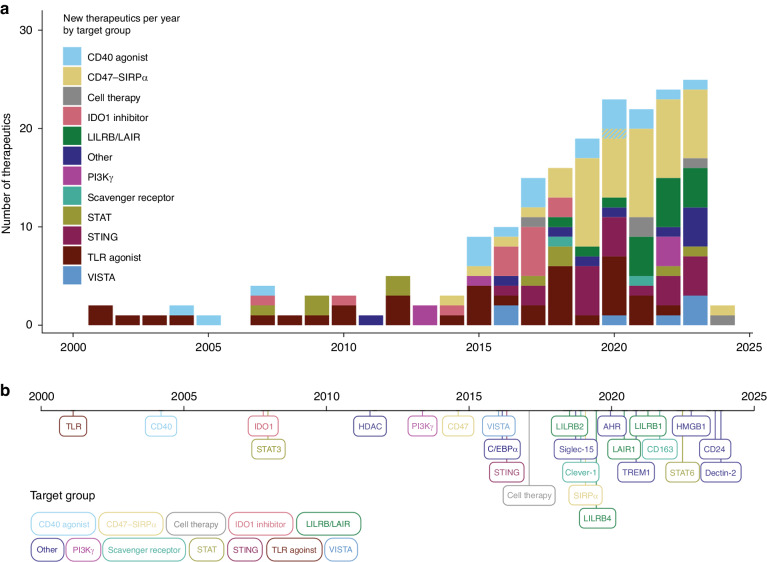
Timeline of clinically evaluated macrophage-reprogramming therapeutics. **a** Number of macrophage-reprogramming therapeutics entering clinical trials each year since 2000, coloured by target group. **b** A timeline showing when each macrophage-reprogramming therapeutic target was first clinically investigated in cancer. Bubble colours indicate macrophage target groups.

Overall, the past decade has seen a steep increase in the number of TAM-reprogramming therapeutics that have entered clinical trials (Fig. 1a). Therapeutics targeting the CD47–SIRPα axis have particularly contributed to this rapid development. Additionally, several novel macrophage targets have entered the clinics, including members of the LILRB and scavenger receptor families (Fig. 1b).

**Fig. 2:**
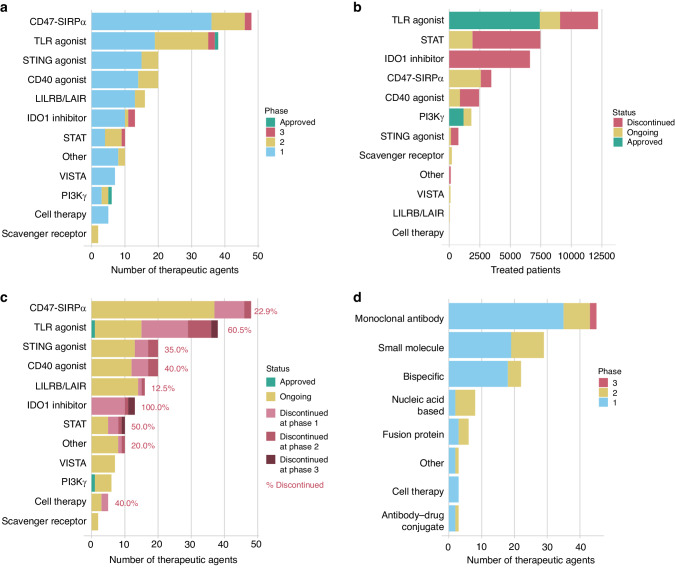
Clinical landscape of macrophage-reprogramming therapeutics in cancer. **a** Number of therapeutic agents that have been investigated in clinical trials. Therapeutics are shown by target group and coloured by clinical development phase as of January 2024. **b** Number of treated patients by target group, coloured by the development status. **c** Number of therapeutic agents by target group, coloured by development status and highest clinical development phase at the time of discontinuation. Percentages indicate proportions of discontinued therapeutics. **d** Bar plots of macrophage-reprogramming therapeutics with ongoing clinical development as of January 2024, grouped by molecule type and coloured by clinical development phase.

Such development is encouraging because currently only a few macrophage-modifying therapies have been approved for clinical use, the number of therapeutics against the same target is high, and not many programmes have proceeded beyond phase 2 (Fig. 2a). The approved therapeutics are duvelisib, a PI3Kγ and PI3Kδ inhibitor for haematological malignancies [53], and imiquimod, a toll-like receptor 7 (TLR7) agonist for topical treatment of basal cell carcinoma [54]. Additionally, approved early activators of pattern recognition receptors include Bacillus Calmette-Guerin, an attenuated bacteria that activates TLR2 and TLR4 receptors in non-invasive bladder cancer [54], and mifamurtide, a synthetic analogue of bacterial cell wall that activates TLR4 and NOD2 receptors in osteosarcoma [55].

Phase 3-investigated therapeutics comprise additional TLR agonists, CD47–SIRPα axis blockers, IDO1 inhibitors and STAT inhibitors (Fig. 2a), and we report staggeringly high numbers of patients treated with these agents (Fig. 2b). Unfortunately, these advanced targets show a high proportion of discontinued therapeutics, such as 100% for IDO1, 61% for TLRs and 50% for STAT, indicating non-favourable therapeutic properties (Fig. 2c). Surprisingly, some targets with known safety- or efficacy-related issues are still highly investigated, such as CD47–SIRPα axis, STING and CD40 (Fig. 2c), reflecting novel approaches taken to develop next-generation agents with a more favourable therapeutic profile. We will review the properties of each target separately further below, but commonly poor efficacy is caused by on-target side effects that limit dosing, or activation of compensatory/counteracting pathways that enable tumour immune escape.

Emerging efficacy- and safety-related challenges have motivated a vast amount of translational research. Novel treatment strategies have already been clinically investigated alongside conventional small molecules and monoclonal antibodies (Fig. 2d), and various approaches have been exploited to circumvent the original challenges. To direct treatment effects, alternative delivery routes (intratumoural vs. systemic) are utilised [56–58]. To target systemically administered therapeutics to the TME, antibody–drug-conjugates [59, 60], antibodies with pH-dependent target binding [61] and bi-specific antibodies and fusion proteins have been developed [56, 62, 63]. Although most TAM-targeted bi-specifics bind a second target on cancer cells or the TME, some use the second arm to enhance immune activation by engaging PD-1, PD-L1 or 4-1BB instead (Supplementary Table 1). The efficacy of monoclonal antibodies can be modified by re-engineering the Fc region when the efficacy depends on antibody-induced effector functions or cross-linking [56, 64]. Indeed, we observed that most of the clinical candidates carry an IgG1 Fc region, which has been occasionally modified to decrease or enhance effector functions (data not shown). Finally, more complex delivery systems using exosomes [65, 66], bacteria [67] or viral vectors [68, 69] have also been tested in clinics (e.g. NCT04592484, NCT05375604, NCT04167137, NCT03852511, NCT02654938). Overall, some of the above approaches have demonstrated superior efficacy or safety in preclinical studies [63, 70, 71], supporting the advancement of their clinical development.

**Fig. 3:**
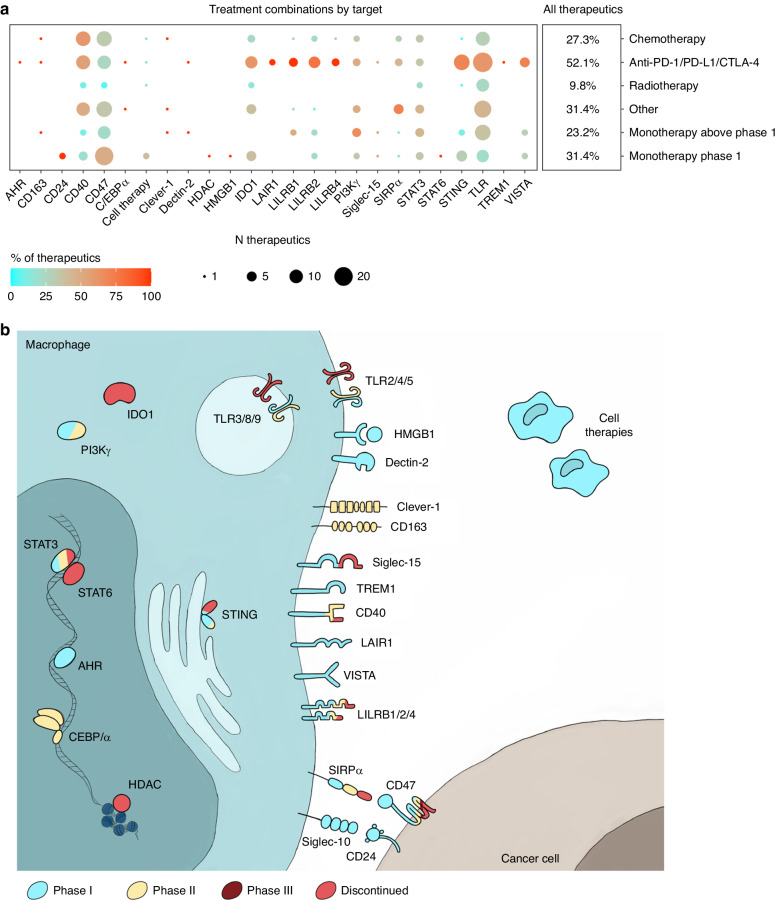
TAM-reprogramming treatment targets by combinatory treatment regimen and subcellular localisation. **a** Dot plot showing how commonly macrophage-reprogramming therapeutics have been investigated in combination with other treatment types. Dot size indicates number and dot colour proportion of macrophage-reprogramming therapeutics investigated with the indicated treatment combinations, separately for each target. ‘Monotherapy phase 1’ indicates therapeutics at phase 1 clinical development not yet investigated in combination with other treatments. Percentages were calculated from all TAM-reprogramming therapeutics (n = 194). **b** Illustration depicting subcellular localisation of macrophage-reprogramming therapeutic targets. Targets are coloured by clinical development status with coloured areas depicting proportions among therapeutics against the same target.

Because TAM-targeted therapeutics are predicted to support the efficacy of other treatments rather than eradicate cancer on their own [4, 5], we also evaluated the prevalence of combination treatment regimens. Expectedly, less than a quarter of clinically investigated TAM-reprogramming agents have been studied as monotherapy above phase 1, whereas more than half have been studied in combination with ICIs (Fig. 3a).

To summarise, a surge of macrophage-reprogramming therapeutics has proceeded to clinical development during the past decade, and while several older targets have faced challenges, novel strategies have been undertaken to improve their therapeutic profiles.

## TAM-reprogramming therapeutic targets

Next, we will discuss each TAM-reprogramming target separately, focussing on the mode-of-action, types of clinically investigated therapeutics and clinical results thus far. Firstly, we describe strategies that alter specific TAM functions, such as phagocytosis, scavenging, pattern recognition or interactions with other immune cells, and then proceed to therapeutic strategies that alter the overall TAM phenotype towards pro-inflammatory direction. Subcellular localisation of each target is illustrated in Fig. 3b. As the majority of these therapeutics have been evaluated in (advanced) solid tumours and haematological malignancies, we will only occasionally describe the investigated cancer types and provide a supplementary trial ID table (Supplementary Table 1) for further reference.

### Phagocytosis checkpoints

#### CD47–SIRPα

To escape phagocytosis, healthy and cancerous cells express CD47, which binds macrophage SIRPα to inhibit cytoskeletal rearrangements necessary for phagocytosis [72, 73]. Since the first-in-class monoclonal CD47-targeting antibody magrolimab [74], ~50 therapeutics blocking the CD47–SIRPα interaction have been clinically investigated. A notable portion of these therapeutics are bi-specific antibodies and fusion proteins that mostly recognise another molecule on cancer cells or inhibit PD-L1/PD-1. Overall, blocking CD47 in haematological cancers and SIRPα in solid tumours yields better efficacy [75], but two phase 3 magrolimab trials (NCT4313881 and NCT04778397) were recently terminated because of poor efficacy in acute myeloid leukaemia and myelodysplastic syndrome [76, 77]. CD47 blockade on red blood cells prevents the therapeutic from reaching cancer cells and causes anaemia by inducing red blood cell phagocytosis and agglutination. To circumvent this, newer strategies use antibodies that preferentially bind to cancer cells or bi-specific agents to target cancer cells [63, 75]. Furthermore, enhanced phagocytosis can promote either pro- or anti-inflammatory reprogramming of TAMs, depending on signals from the phagocytosed cells and type of phagocytosis. Anti-inflammatory responses would limit monotherapy efficacy by promoting immunosuppression and tumour growth [78].

#### CD24–Siglec-10

Analogous to CD47–SIRPα interaction, CD24 on cancer cells inhibits phagocytosis by binding to macrophage Siglec-10 [79]. CD24-targeting antibodies of the IgG1 subclass (IMM47, ATG-031) are investigated in phase 1 trials, and at least preclinical IMM47 efficacy depends on its Fc-induced effector functions [80]. Further development should consider other immune- and cancer-related functions of CD24 and additional CD24 and Siglec-10 ligands binding to different glycosylated protein forms [79, 81, 82].

### Scavenger receptors

Macrophages clear various endogenous and pathogen-related ligands with scavenger receptors that also regulate subsequent immune responses to these ligands [83, 84]. Apart from clinically investigated CD163 and Clever-1, preclinical studies have identified MARCO [85, 86] and CD206 [87] as potential therapeutic targets. Inhibiting MARCO with the monoclonal antibody PY265 supports anti-tumour immunity via pro-inflammatory conversion of TAMs and MDSCs [88], and specific CD206-targeted peptides can likewise support M1-like macrophage phenotype or deliver therapeutics to CD206-expressing TAMs [87, 89].

#### CD163

Under homoeostasis, CD163 scavenges haptoglobin–haemoglobin complexes [90], and in cancer, CD163 is a widely known marker for tumour-promoting M2-like macrophages, generally associated with poor prognosis [83, 91, 92]. CD163 in TAMs is associated with STAT3 activation and anti-inflammatory IL-10 and TGF-β secretion [93]. A single CD163-targeting antibody (OR2805, IgG1 subclass), which activates T-cell responses in preclinical models [94], is being clinically evaluated in a phase 1/2 trial (NCT05094804).

#### Clever-1

Clever-1 (Stabilin-1) is a scavenger and adhesion molecule expressed by monocytes, macrophages and endothelial cells [95]. It regulates macrophage lysosomal acidification to halt antigen presentation and T-cell activation [96]. A Clever-1-blocking antibody, bexmarilimab (IgG4), activates T-cells and IFN responses in advanced solid cancers and elicits cancer type-dependent disease control alongside few objective responses in a phase 1/2 trial [96, 97]. Promising objective responses have been observed in combination with azacytidine in acute myeloid leukaemia and myelodysplastic syndrome in a phase 1/2 trial (NCT05428969) that is also recruiting azacytidine-refractory patients [98].

### Pattern recognition

Pattern recognition receptors alert innate immunity to pathogens and tissue damage, but their systemic administration is often limited by the associated side effects of overt immune activation [99].

#### Toll-like receptors (TLRs)

TLRs are widely expressed, but their stimulation on antigen-presenting cells activates pro-inflammatory cytokine secretion, expression of co-stimulatory molecules and antigen presentation to support T-cell activation [100–103]. Depending on the TLR type, the activating therapeutics are either small molecules or larger lipid or nucleic acid derivatives that can be administered systemically or locally [58, 100]. Several therapeutics have faced discontinuation after showing weak monotherapy efficacy [101], possibly due to the induction of tolerance and simultaneous activation of pro-tumoural pathways that support regulatory T cells (Tregs), MDSCs and cancer cell proliferation [104–106]. Mostly, these agents are now deemed as boosters for other therapeutics, especially cancer vaccines and ICIs [102]. As an exception, the TLR7 agonist imiquimod and the attenuated TLR2- and TLR4-activating Bacillus Calmette-Guerin bacteria have been approved for skin carcinoma and non-muscle invasive bladder carcinoma, respectively [54].

#### STING

Upon recognising cytosolic pathogen-derived or damaged endogenous DNA, cGAS produces cyclic GMP–AMP, which activates STING to elicit type I IFN production and NF-κB activation. These pathways promote cancer cell death, anti-inflammatory macrophage polarisation, antigen presentation, T-cell priming and recruitment [107, 108]. However, chronic STING activation may have undesired opposite effects, such as enhanced cancer cell survival and immunosuppressive IDO1 induction [108]. Nevertheless, at least twenty STING-activating therapeutics have been clinically evaluated in phase 1 and 2 trials. Early STING agonists were intratumourally administered synthetic cyclic dinucleotides (e.g. ADU-S100, MK-1454) characterised by insufficient monotherapy efficacy, limited penetration inside the cells and susceptibility to enzymatic degradation [57, 109, 110]. Therefore, alternative delivery methods, such as liposomal formulations, nanoparticles, antibody–drug-conjugates (TAK-500, XMT-2056), exosomes (exoSTING: NCT04592484) and a bacterial vector (SYNB1891: NCT4167137) are being developed [109]. Additionally, compounds that enable intravenous administration (SB11285: NCT04096638) and a polymer that prolongs STING activation (ONM-501: NCT06022029) are under clinical investigation.

#### HMGB1

As a DNA-binding protein, HMGB1 supports chromatin organisation and transcription, but extracellular HMGB1 released from dying cells and activated macrophages stimulates inflammatory responses via TLRs and RAGE [111, 112]. In cancer, some HMGB1-activated pathways aggravate prognosis by supporting invasion and metastasis [112, 113]. The HMGB1-binding prodrug SB17170 modulates myeloid cell cytokine secretion to increase T-cell infiltration [114], and it is being evaluated in a phase 1 trial (NCT05522868).

#### Dectin-2

Dectin-2 (CLEC6A) defends against fungi and mycobacteria [115, 116]. Its activation stimulates the secretion of pro-inflammatory chemokines and cytokines, including TNFα and IL-12 [116, 117], and the receptor enables liver-resident Kupffer cells to recognise and phagocytose cancer cells [118]. The agonistic antibody BDC-3042 preferentially binds to TAMs in patient tumour samples [117], and it is being investigated in a phase 1/2 trial as a monotherapy and with pembrolizumab (NCT06052852).

### Co-stimulatory and inhibitory receptors

#### CD40

Antigen-presenting cells express CD40 that, upon engagement by CD40L on activated T helper cells, upregulates the expression of pro-inflammatory cytokines, MHC and co-stimulatory molecules crucial for T-cell-mediated immune responses [56, 119]. We report 20 CD40-activating phase 1- or phase 2-investigated therapeutics, most commonly agonistic monoclonal antibodies, or bi-specific antibodies and fusion proteins. Several therapeutics have been discontinued because of modest efficacy and dose-limiting toxicities, such as cytokine release syndrome, liver toxicity and enhanced angiogenesis [56]. The safety profile can be improved with bi-specific agents additionally targeting the TME (NCT02955251 for mesothelin; NCT05740202, NCT05740202 and NCT05098405 for fibroblast activation protein alpha), but these agents have yet to show objective anti-tumour responses [62, 120, 121]. To enhance potency, the Fc region of some antibodies has been re-engineered to potentiate crosslinking (2141-V11 and sotigalimab) [56, 64]. Sotigalimab combined with anti-PD-1 treatment, and another optimised CD40-targeting antibody, mitazalimab, combined with chemotherapy have enhanced objective response rates in anti-PD-1-resistant melanoma and pancreatic cancer, respectively [122, 123].

#### LILRB family

The LILRB family receptors LILRB1, LILRB2 and LILRB4 bind various ligands to promote tolerogenic and immunosuppressive functions [124]. For instance, macrophage LILRB1 suppresses phagocytosis upon binding to MHC-I molecules on cancer cells [125], and antagonising macrophage LILRB2 promotes pro-inflammatory cytokine secretion [126]. In cancer, both immune and cancer cells may express these receptors [124]. Several antagonistic antibodies against LILRB family members, most commonly LILRB2, are being clinically investigated. The first-in-class MK-4830 has demonstrated durable objective responses along with pembrolizumab, even in patients previously progressing on anti-PD-1/PD-L1 therapy [127]. Newer antagonistic antibodies, IO-202 (LILRB4) and IO-108 (LILRB2), have elicited a few objective responses as mono- or combination therapies in haematological malignancies and solid tumours, respectively [128, 129].

#### LAIR1

The widely expressed collagen-binding and immune-inhibitory receptor LAIR1 plays both cancer-promoting and cancer-inhibiting roles [130]. In solid malignancies, immunosuppression results from myeloid LAIR1 binding collagen, which can be prevented with a LAIR1 antagonist (NGM438) [131] or a LAIR2 fusion protein (NC410) that competes for the same collagen ligands [132, 133]. Unfortunately, LAIR1 inhibition may also enhance metastasis [134], and early clinical results suggest weak NC410 monotherapy efficacy (NCT04408599).

#### Siglec-15

Siglec-15 resembles PD-L1 in its extracellular domain structure, ability to suppress T-cell responses and elevated expression in the TME. In contrast to PD-L1, IFNγ downregulates Siglec-15, resulting in differing expression patterns [135]. Macrophage Siglec-15 induces TGF-β secretion and directly downregulates NF-κB/NFAT signalling in T cells [135, 136]. Despite initial objective responses, low efficacy has led to discontinuation of NC318 monotherapy in phase 2 [137, 138]. Another Siglec-15-blocking antibody, PYX-106, is presently in phase 1 (NCT05718557).

#### TREM1

Immunosuppressive cytokines and hypoxia upregulate TREM1 expression in myeloid cells, and its activation amplifies inflammatory responses [139, 140]. Activating monocyte/neutrophil TREM1 with the agonistic antibody PY159 (IgG1) enhances co-stimulatory molecule expression and IFNγ responses to support anti-tumour immunity [139], but TREM1 is also associated with poor prognosis and inflammation-related carcinogenesis [139, 141]. PY159 has been evaluated in a phase 1 trial as a monotherapy and in combination with pembrolizumab (NCT04682431). Another TREM family member, TREM2, sustains chronic inflammation in immunosuppressive TAM subsets [142, 143]. Current clinical development focuses on depleting TREM2-expressing TAMs [144], and is therefore outside the scope of this review. However, strategies antagonising TREM2 function without TAM depletion are also being preclinically developed [142, 143].

#### VISTA

VISTA is a B7 family immune checkpoint molecule upregulated by inflammatory stimuli and is most highly expressed on myeloid cells. Both T-cell and myeloid VISTA limit T-cell responses, for instance, by downregulating T-cell receptor signalling and promoting Treg phenotype [145]. Several monoclonal VISTA-blocking antibodies and one oral B7 ligand inhibitor (CA-170) are evaluated in phase 1/2 trials. Some of the antibodies activate TAMs and CD8^+^ T cells without Fc-mediated effector functions (HMBD-002: NCT05082610) [146], whereas the efficacy of others depends on the IgG1 subclass (CI-8993: NCT04475523; KVA12123: NCT05708950) [147, 148]. pH-sensitive antibodies have been designed to preferentially target VISTA in the acidic TME rather than on circulating immune cells [61].

### Metabolic enzymes

#### IDO1

Along with other tryptophan-catabolising enzymes (TDO, IL4I1, IDO2), IDO1 converts tryptophan amino acids into kynurenine metabolites. Both tryptophan depletion and kynurenine-activated AHR transcription factors limit anti-tumour immunity by restricting T-cell activation, inducing Tregs and promoting myeloid cell tolerance. Cancer cells can constitutively express IDO1, but IDO1 on immune cells is IFN inducible [149, 150]. We report 13 clinically investigated small molecule inhibitors of IDO1 or IDO1 and TDO2. The most advanced clinical candidates, epacadostat (e.g. NCT02752074, NCT03260894) and linrodostat (NCT03329846), have been investigated in several phase 3 trials. However, after epacadostat failed to improve survival along with pembrolizumab in a large phase 3 melanoma trial [151], IDO1-targeting programmes have been discontinued. The low efficacy may be explained by immunotherapy-induced IFNγ upregulating IDO1 and compensatory enzymatic activity by other enzymes [149, 150]. Some IDO1 inhibitors also counteractively bind to and activate AHR or may not sufficiently penetrate inside the cells within the TME [149, 152].

#### Arginase 1

Arginase 1 catabolizes L-arginine into L-ornithine, resulting in arginine depletion that limits T-cell activation and promotes immunosuppression [153]. While arginase 1 is expressed on immunosuppressive murine macrophages [153, 154], its expression on human macrophages varies based on macrophage source (monocyte-derived vs. tissue-resident) and inflammatory environment [154]. As arginase 1 is expressed on neutrophils and MDSCs rather than TAMs in human malignancies [155–158], small molecule arginase inhibitors (INCB001158, OATD-02) were excluded from our clinical landscape analysis.

### Transcription factors

#### STAT

In TAMs and MDSCs, STAT3 and STAT6 regulate immunosuppressive transcriptional programmes in response to IL-10, VEGF and IL-6, or IL-4 and IL-13, respectively. Additionally, they govern a wide array of pro-tumoural processes in cancer cells and other immune and non-immune cells [159, 160]. At least nine STAT3 inhibitors and one STAT6 inhibitor have been clinically investigated, but the development of several small molecule STAT3 inhibitors has been abandoned because of weak efficacy or intolerable side effects (e.g. NCT02993731, NCT02058017). Alternative STAT-inhibiting strategies utilise antisense oligonucleotides (danvatirsen for STAT3, exoASO-STAT6 for STAT6) or small interfering RNAs (DCR-STAT3: NCT06098651) [65, 161]. For instance, danvatirsen supports macrophage pro-inflammatory responses and inhibits cancer cell proliferation, and it has elicited objective responses in diffuse large B-cell lymphoma [161–163].

#### C/EBPα

The tumour suppressor protein C/EBPα impedes myeloid cell differentiation and MDSC-regulated immunosuppression [164]. To elevate C/EBPα expression, patients with hepatocellular carcinoma have been administered nanoparticle-packaged small activating RNAs (MTL-CEBPA) that promote C/EBPα transcription. The treatment primes these tumours for subsequent kinase inhibitor (sorafenib) treatment to elicit otherwise uncommon objective responses [165, 166]. Curiously, MTL-CEBPA decreases both immunosuppressive MDSCs and pro-inflammatory cytokine secretion, resulting in a net effect of T-cell activation [165, 167].

#### Aryl hydrocarbon receptor (AHR)

AHR is a ligand-activated transcription factor that mediates the immunosuppressive effects of IDO1/TDO-produced kynurenine metabolites, regulates transcriptional programmes in immune cells and affects cancer cell proliferation [168, 169]. The small molecule AHR inhibitor IK-175 increases pro-inflammatory macrophages and activates CD8^+^ T cells in tumour-draining lymph nodes [170]. It has been investigated in a phase 1 trial with nivolumab (NCT04200963), where a few objective monotherapy and combination therapy responses have been reported [171].

### Histone deacetylase (HDAC)

HDAC family enzymes deacetylate proteins, including histones, and thus epigenetically regulate gene expression [172]. While anti-proliferative small molecule inhibitors blocking several HDACs have been approved for treating haematological malignancies [173], one macrophage-directed HDAC inhibitor has been clinically evaluated [174]. More recently, class IIa HDACs have been recognised as putative targets for inducing immunostimulation and phagocytosis in TAMs [175]; however, the complex functions of HDACs in immune cells warrant further studies [176].

### PI3Kγ

The predominantly leucocyte-expressed kinases PI3Kγ and PI3Kδ regulate various downstream signalling pathways and cellular processes [177]. PI3Kγ-disruption supports MHC-II and pro-inflammatory mediator expression on macrophages to strengthen T-cell-mediated anti-tumour responses [178], while PI3Kδ inhibition limits Treg and possibly MDSC functions [179]. One dual PI3Kγ/δ inhibitor, duvelisib, has been approved for haematological malignancies [53], and both dual (NCT05269940, NCT06189209) and PI3Kγ-specific (NCT03961698, NCT05118841, NCT05759234) small molecule inhibitors are being investigated in phase 1 and 2 trials. The first PI3Kγ-specific inhibitor, eganelisib, has shown modest monotherapy efficacy and occasional objective responses along with nivolumab in anti-PD-1/PD-L1-resistant patients [180].

### Cell-based therapies

Early studies administering IFNγ-activated macrophages showed limited efficacy, but advances in macrophage engineering have renewed interest for cell-based strategies [181, 182]. For instance, chimeric antigen receptors (CAR) recognising specific antigens on tumour cells have been added in GM-CSF-differentiated patient macrophages (CT-0508 against HER2) or monocytes (CT-0525 against HER2, MT-101 against CD5) to enhance cancer cell phagocytosis [183, 184]. These therapies are currently investigated in early clinical trials (NCT04660929, NCT06254807, NCT05138458). In comparison to CAR T cells, CAR macrophages and monocytes are predicted to penetrate into solid tumours more efficiently and upon activation modulate the immunosuppressive TME via enhanced antigen presentation, phagocytosis and pro-inflammatory mediators, but their production has been more complicated due to monocyte/macrophage resistance to several transduction methods and limited ex vivo expansion [181, 185]. The selected transduction method and cell source will also affect the resulting macrophage phenotype [185]. Therapeutic efficacy will require sufficient tumour-homing, ability to maintain anti-tumoural phenotype and present antigens to overcome the limitation of recognising a single tumour antigen.

Other clinically investigated approaches include pro-inflammatory macrophages modified with a cytokine cocktail to downregulate and stimulate cancer cell phagocytosis (SIRPant-M) [186] and an intraperitoneally injected autologous monocyte and IFN infusion [187].

## Challenges in TAM-reprogramming: reaching the full potential

The biggest hurdle in transforming preclinically studied treatments into clinically active therapeutics has been the lack of efficacy. While reviewing the clinical trial literature, we observed that several therapeutics induced few partial or complete responses as monotherapy, resulting in insufficiently low overall response rates (~5%) [127, 137, 166, 180]. These could be elevated by identifying biomarkers for eligible patients and determining optimal cancer types, disease stages and treatment regimens. Whether choosing a specific therapeutic is crucial for a certain cancer type or whether the same patients would have favourable responses to several TAM-reprogramming agents is an important question that might relate to targeted TAM subsets. The future will tell whether monotherapy response rates can be sufficiently elevated by optimising patient selection.

Presently, a plethora of therapeutics is directed at the same targets (Fig. 2a), unnecessarily increasing the number of treated patients and development costs for similar approaches. Ideally, novel therapeutics would be compared against other similar treatments earlier, and data on clinically investigated candidates would be more readily accessible. For evaluating therapy-elicited anti-tumoural immune responses, patient-derived ex vivo tumour models are valuable platforms because they can provide predictive information on clinical responses [188]. Measured parameters should be carefully selected because not all pro-inflammatory mediators correlate with treatment benefit and many have context-dependent effects. For instance, the pro-inflammatory cytokine IL-1β and immune-activating damage-associated molecule HMGB1 can promote cancer development and progression [113, 189, 190]. Even IFNγ, which mediates critical steps of anti-tumoural immune responses, can, depending on signalling length and strength, promote tumour growth instead [191]. This complexity of immune responses in the TME calls for the evaluation of the whole system rather than single cytokines.

Preferably, some developmental efforts should be re-directed for searching novel macrophage-specific treatment strategies and expanding our understanding of TAM biology upon therapeutic intervention. Conducted trials have highlighted several important features for suitable treatment targets, including the ability to elicit an appropriate level of immune activation without overtly activating counteractive pathways or negative feedback mechanisms [105, 106, 152, 192], tendency to alter TAMs rather than healthy tissue macrophages [193], lack of compensatory pathways in the TME [149] and access for therapeutic manipulation within solid tumours [57]. To direct therapeutic effects away from macrophages in healthy tissues, therapeutic efficacy could depend on tumour-specific expression, ongoing chronic inflammation at tumour sites or monocyte-derived macrophages. Enhancing specificity with such TME-specific targets or TME-directed delivery mechanisms could potentially widen the therapeutic window that is currently limited by side effects [22, 56, 63, 194]. Alternatively, targeting circulating monocytes can affect their differentiation into TAMs and therefore provide access within solid tumours without the need for direct tissue penetration.

Fundamental understanding of TAM biology will pave the way to more effective treatment approaches [4, 5, 195]. Unfortunately, TAM subset complexity revealed by single-cell RNA sequencing [1, 10, 45] is not yet accommodated by clinically investigated therapeutics. Furthermore, orthotopic murine tumour models used in preclinical studies are unable to recapitulate such intricate TAM biology [196]. As these newly identified TAM states are suggested to possess both anti- and pro-tumoural functionalities within the same cell subsets [10], removing pro-tumoural TAMs would be extremely challenging. Instead, careful and possibly subset-specific modulation is required to support their anti-tumoural functions. The plastic nature of macrophages should allow this type of modulation, but TAM functions are also highly tied to their intratumoural localisation [10, 11, 196–198]. Therefore, therapeutic manipulation must overcome the TME-imposed restriction of TAM responses to sufficiently change their behaviour. The efforts to reprogramme TAMs should be combined with approaches that deeply probe the treatment effects on different TAM subsets and their interactions.

Finally, combinatory strategies have been predicted to be central for TAM-targeted therapy efficacy [4, 5], but therapeutic combinations and their timing need to be carefully selected, as TAMs affect the efficacy of other cancer therapies and TAM phenotypes change over cancer progression [4, 5, 199]. Dual effects on the efficacy of chemotherapy, radiotherapy and immunotherapy have been reported, and several excellent reviews discuss these interactions [5, 200, 201]. Importantly, chemo- and radiotherapy can either support pro-inflammatory TAM polarisation and tumour cell killing [201–203] or induce tissue repair responses associated with anti-inflammatory TAM polarisation and immunosuppression [201, 204, 205], depending on chemotherapeutic agent or radiation dose [5, 200]. Direct depletion of monocytes or TAMs can also significantly contribute to the anti-tumoural efficacy of chemotherapy, as has been shown for trabectedin [206]. While the efficacy of several monoclonal antibodies depends on macrophage-mediated effector functions and activation of anti-tumour immunity, TAM-regulated immunosuppression and T-cell exclusion remain major obstacles for effective immunotherapy [26, 207–209]. Successful combinatory treatment regimens thus require mechanism-based and data-driven designs.

Of particular note, combining ICIs with TAM-targeted therapeutics can provide synergistic benefits because removing TAM-mediated restraint of T-cell responses can sensitise the tumour for further ICI therapy [5, 210]. More information on treatment-induced changes in TAMs and the surrounding TME may also expose vulnerabilities that can be attacked with other therapeutics [4]. For instance, CSF-R1 blockade in murine glioma leads to treatment resistance via upregulation of IGF-1 and PI3K signalling, which can be prevented with PI3K inhibitor treatment [211].

To use the full potential of TAM-reprogramming agents, research-driven approaches are needed to solve these challenges and develop a next generation of more effective treatment strategies (Table 1). Although TAM targeting has proven to be a difficult task, TAMs are evidently a key component of the TME and should not be overlooked to make significant advances for treating cancer.

**Table 1:** Emerging challenges in developing effective TAM-reprogramming therapeutics and potential solutions or development opportunities for reaching the full therapeutic potential of targeting TAMs.

Challenge	Development opportunity
Poor monotherapy efficacy	Identify biomarkers for eligible patients
	Select the optimal patient population (cancer type, disease stage, treatment regimen)
	Combine with other therapeutics
Combinatory approaches complicated by the dual effects of other therapeutics on TAMs	Design data-driven combinatory treatment regimens that consider therapy-induced changes in TAMs
Low specificity for TAMs limits dosing and narrows the therapeutic window	Identify TAM-specific targets to reduce side effects
	Use TME-targeted therapeutics or delivery strategies
Overlapping or redundant clinical trials	Compare therapeutics earlier using ex vivo, in vivo or in vitro models
	Improve data access for ongoing and discontinued development projects
	Identify novel targets
Incomplete understanding of TAM biology	Correlate therapeutic responses with TAM subsets and localisation at pre-treatment
	Perform high-dimensional analyses of changes in TAM subsets upon therapeutic intervention
	Identify TAM subset vulnerabilities and therapy-induced changes for novel therapeutic strategies
Dual roles of inflammatory mediators in cancer progression	Evaluate immune responses at the system level rather than individual cytokines

## Conclusions

All in all, past and present trials have proven that complete responses are occasionally attainable with TAM-reprogramming monotherapy, although wider benefits will first be achieved in combination with other treatment modalities. The path forward should emphasise selected data-driven treatment strategies that use state-of-the-art knowledge on TAM biology and patient biomarkers.

### Supplementary information

Supplemental Material

## Data Availability

A Supplementary table of collected therapeutics and corresponding trial IDs can be accessed via the electronic version of the manuscript (Supplementary Table 1).
