# Supplementary material for: Clinical landscape of macrophage-reprogramming cancer immunotherapies
Source: Br J Cancer. 2024 Jun 3;131(4):627–40. doi: 10.1038/s41416-024-02715-6 (PMC11333586; doi:10.1038/s41416-024-02715-6)
Supplement: Supplementary file 1 — Supplemental Material [file 41416_2024_2715_MOESM1_ESM.pdf]

Supplementary Table 1. A list of cancer immunotherapeutic agents that reprogram macrophage function or phenotype.

Target	Therapeutic*	Molecule type	Phase**	Trial ID
**AHR**	IK-175	Small molecule	1	NCT04200963, NCT05472506
**CD163**	OR2805	mAb	1/2	NCT05094804
**CD24**	IMM47	mAb	1	NCT05985083
**CD24**	ATG-031	mAb	1	NCT06028373
**CD40**	Dacetuzumab; SGN-40	mAb	2	NCT00079716, NCT00103779, NCT00283101, NCT00435916, NCT00525447, NCT00529503, NCT00556699, NCT00655837, NCT00664898
**CD40**	Selicrelumab; CP-870,893; RO7009789	mAb	1/2	NCT02157831, NCT01008527, NCT01456585, NCT02304393, NCT02588443, NCT02665416, NCT02760797, NCT03193190, NCT03424005, NCT03555149, NCT03892525
**CD40**	ChiLob 7/4	mAb	1	NCT01561911
**CD40**	SEA-CD40	mAb	2	NCT02376699, NCT04993677
**CD40**	Mitazalimab; ADC-1013; JNJ-64457107	mAb	1/2	NCT02379741, NCT02829099, NCT05650918, NCT04888312, NCT06205849
**CD40**	Sotigalimab; APX005M; PYX-107	mAb	2	NCT02482168, NCT02600949, NCT02706353, NCT03123783, NCT03214250, NCT03165994, NCT03389802, NCT03502330, NCT03597282, NCT03719430, NCT04337931, NCT04130854, NCT04495257, NCT05419479
**CD40**	ABBV-428	Bispecific Ab (Mesothelin)	1	NCT02955251
**CD40**	ABBV-927	mAb	1/2	NCT02988960, NCT03893955, NCT03818542, NCT04807972
**CD40**	MEDI5083	Fusion protein	1	NCT03089645
**CD40**	CDX-1140	mAb	2	NCT03329950, NCT04491084, NCT04536077, NCT04520711, NCT05029999, NCT05484011, NCT04616248, NCT05349890, NCT05849480, NCT05231122
**CD40**	NG-350A	Viral vector for mAb	1	NCT03852511, NCT04787991, NCT05165433
**CD40**	GEN1042; BNT312	Bispecific Ab (4-1BB)	1/2	NCT04083599, NCT05491317, NCT06057038
**CD40**	2141 V-11	mAb	1/2	NCT04059588, NCT04547777, NCT05126472, NCT05734560
**CD40**	YH003	mAb	2	NCT04481009, NCT05017623, NCT05031494, NCT05176509, NCT05420324
**CD40**	LVGN7409	mAb	1	NCT04635995, NCT05152212, NCT05075993
**CD40**	RO7300490; RG6189	Bispecific Ab (FAP)	1	NCT04857138

**CD40**	MP0317	Bispecific protein (FAP)	1	NCT05098405
**CD40**	MIL-97	mAb	1	NCT04965077
**CD40**	SHR-7367	Bispecific (FAP)	1	NCT05740202
**CD40; CD47**	SL-172154	Bispecific protein	1	NCT04406623, NCT04502888, NCT05275439, NCT05483933
**CD47**	Magrolimab; Hu5F9-G4	mAb	3	NCT02216409, NCT02678338, NCT02953782, NCT02953509, NCT03248479, NCT03558139, NCT03527147, NCT03869190, NCT03922477, NCT04435691, NCT04313881, NCT04541017, NCT04778410, NCT04778397, NCT04751383, NCT04854499, NCT04827576, NCT04892446, NCT04958785, NCT04599634, NCT05169944, NCT04788043, NCT05079230, NCT05330429, NCT05738161, NCT05835011, NCT06046482, NCT05829434, NCT05807126, NCT05367401
**CD47**	CC-90002	mAb	1	NCT02367196, NCT02641002
**CD47**	Ontorpacept; TTI-621; PF-0791800	Fusion protein	2	NCT02663518, NCT02890368, NCT04996004, NCT05507541
**CD47**	Evorpacept; ALX148	Fusion protein	2/3	NCT03013218, NCT04417517, NCT04643002, NCT04675294, NCT04755244, NCT05027139, NCT05025800, NCT05002127, NCT05167409, NCT05524545, NCT05467670, NCT05868226, NCT05787639, NCT04675333
**CD47**	SRF231	mAb	1	NCT03512340
**CD47**	Maplirpacept; PF-07901801; TTI-622	Fusion protein	2	NCT03530683, NCT05139225, NCT05261490, NCT05567887, NCT05675449, NCT05507541, NCT05896774, NCT05626322, NCT05896163
**CD47**	SHR1603	mAb	1	NCT03722186
**CD47**	IBI188	mAb	1	NCT03717103, NCT03763149, NCT04485052, NCT04485065, NCT04861948
**CD47**	AO-176	mAb	1/2	NCT03834948, NCT04445701
**CD47**	TG-1801	Bispecific Ab (CD19)	1	NCT03804996, NCT04806035
**CD47**	lemzoparlimab; TJ011133	mAb	3	NCT03934814, NCT04202003, NCT04912063, NCT05148533, NCT04895410, NCT05709093
**CD47**	HX009	Bispecific Ab (PD-1)	2	NCT04097769, NCT05731752, NCT04886271, NCT05189093
**CD47**	SGN-CD47M	Antibody–drug conjugate	1	NCT03957096
**CD47**	IMM01	Fusion protein	1/2	NCT05860075, NCT05140811, NCT05833984
**CD47**	IMM0306	Bispecific protein (CD20)	1/2	NCT05805943, NCT04746131, NCT05771883
**CD47**	ZL-1201	mAb	1	NCT04257617

**CD47**	IMC-002	mAb	1	NCT04306224, NCT05276310, NCT05946226
**CD47**	IBI322	Bispecific Ab (PD-L1)	2	NCT04328831, NCT04338659, NCT04795128, NCT04912466, NCT05296603, NCT05148442
**CD47**	DSP107	Bispecific protein (4-1BB)	1/2	NCT04440735, NCT04937166
**CD47**	MIL-95	mAb	1	NCT04651348
**CD47**	AK117; Ligufalimab	mAb	2	NCT04349969, NCT04728334, NCT04900350, NCT04980885, NCT05214482, NCT05227664, NCT05229497, NCT05235542, NCT05382442, NCT05960955, NCT06196203
**CD47**	Gentulizumab	mAb	1	NCT05263271, NCT05221385
**CD47**	TQB2928	mAb	1	NCT04854681, NCT05192512, NCT06008405
**CD47**	PF-07257876	Bispecific Ab (PD-L1)	1	NCT04881045
**CD47**	IBC0966	Bispecific Ab (PD-L1)	1/2	NCT04980690
**CD47**	6MW3211	Bispecific Ab (PD-L1)	2	NCT05048160, NCT05448599, NCT05431569, NCT05440045, NCT05446688
**CD47**	STI-6643	mAb	1	NCT04900519
**CD47**	CPO107	Bispecific protein (CD20)	1/2	NCT04853329
**CD47**	BAT7104	Bispecific Ab (PD-L1)	1	NCT05767060, NCT05200013
**CD47**	IMM2902	Bispecific protein (HER2)	1/2	NCT05805956, NCT05076591
**CD47**	NI-1801	Bispecific Ab (Mesothelin)	1	NCT05403554
**CD47**	HMPL-A83	mAb	1	NCT05429008
**CD47**	SG2501	Bispecific Ab (CD38)	1	NCT05293912
**CD47**	AUR103	Small molecule	1	NCT05607199
**CD47**	PT886	Bispecific Ab (Claudin 18.2)	1/2	NCT05482893
**CD47**	IMM2520	Bispecific protein (PD-L1)	1	NCT05780307
**CD47**	SG1906	Bispecific Ab (Claudin 18.2)	1	NCT05857332
**CD47**	PT217	Bispecific Ab (DLL3)	1	NCT05652686
**CD47**	D3L-001	Bispecific Ab (HER2)	1	NCT05957536
**CD47**	HCB101	Fusion protein	1	NCT05892718

**C/EBPα**	MTL-CEBPA	Small activating RNA	2	NCT02716012, NCT04105335, NCT05097911, NCT04710641
**Cell therapy**	CT-0508	Macrophage-based cell therapy	1	NCT04660929
**Cell therapy**	CT-0525	Monocyte-based cell therapy	1	NCT06254807
**Cell therapy**	MT-101	Monocyte-based cell therapy	1/2	NCT05138458
**Cell therapy**	SIRPant-M; SI-101	Macrophage-based cell therapy	1	NCT05967416
**Cell therapy**	Autologous monocytes, peginterferon alfa-2b and interferon gamma-1b	Monocyte-based cell therapy	1	NCT02948426
**Clever-1**	Bexmarilimab; FP-1305	mAb	1/2	NCT03733990, NCT05428969
**Dectin-2**	BDC-3042	mAb	1/2	NCT06052852
**HDAC**	Tefinostat; CHR-2845	Small molecule	1/2	NCT00820508, NCT02759601
**HMGB1**	SB17170	Small molecule	1	NCT05522868
**IDO1**	Indoximod; NLG-8189; 1-methyl-D-tryptophan	Small molecule	2	NCT00567931, NCT00739609, NCT01042535, NCT01191216, NCT01560923, NCT01792050, NCT02052648, NCT02073123, NCT02077881, NCT02502708, NCT02460367, NCT02835729, NCT03301636, NCT04049669, NCT05106296
**IDO1**	Epacadostat; INCB024360	Small molecule	3	NCT01195311, NCT01604889, NCT01685255, NCT01822691, NCT01961115, NCT02178722, NCT02118285, NCT02042430, NCT02166905, NCT02298153, NCT02327078, NCT02318277, NCT02575807, NCT02364076, NCT02559492, NCT02785250, NCT02752074, NCT02862457, NCT02959437, NCT03085914, NCT03196232, NCT03277352, NCT03358472, NCT03361865, NCT03260894, NCT03322540, NCT03374488, NCT03348904, NCT03322566, NCT03322384, NCT03414229, NCT03006302, NCT03217669, NCT03291054, NCT03361228, NCT03347123, NCT03463161, NCT03493945, NCT03589651, NCT03602586, NCT03707457, NCT03823131, NCT03471286, NCT03516708, NCT03532295, NCT04463771, NCT04586244
**IDO1**	GDC-0919; navoximod	Small molecule	1	NCT02048709, NCT02471846
**IDO1**	BMS-986205; linrodostat	Small molecule	3	NCT02658890, NCT02750514, NCT02935634, NCT02996110, NCT03192943, NCT03247283, NCT03346837, NCT03329846, NCT03335540, NCT03459222, NCT03519256, NCT03695250,

				NCT03792750, NCT03854032, NCT04047706, NCT04106414
**IDO1**	KHK2455	Small molecule	1	NCT02867007, NCT03915405
**IDO1**	PF-06840003	Small molecule	1	NCT02764151
**IDO1**	NLG802	Small molecule	1	NCT03164603
**IDO1**	LY3381916	Small molecule	1	NCT03343613
**IDO1**	MK-7162	Small molecule	1	NCT03364049
**IDO1; TDO**	HTI 1090; SHR9146	Small molecule	1	NCT03208959, NCT03491631
**IDO1; TDO**	M4112	Small molecule	1	NCT03306420
**IDO1; TDO**	DN1406131	Small molecule	1	NCT03641794
**IDO1; TDO**	LPM-3480226	Small molecule	1	NCT03844438
**LAIR1**	NGM438	mAb	1	NCT05311618
**LAIR1**	NC410	Fusion protein	1/2	NCT04408599, NCT05572684
**LILRB1**	SAR444881; BND-22	mAb	1/2	NCT04717375
**LILRB1**	AGEN1571	mAb	1	NCT05377528
**LILRB2**	MK-4830	mAb	2	NCT03564691, NCT04165070, NCT04165096, NCT04303169, NCT04626518, NCT04165083, NCT04895722, NCT04924101, NCT04541108, NCT04938817, NCT05446870, NCT05342636, NCT05319730
**LILRB2**	JTX-8064	mAb	1/2	NCT04669899
**LILRB2**	IO-108	mAb	1	NCT05054348, NCT05508100
**LILRB2**	BMS-986406	mAb	1	NCT05298592
**LILRB2**	CDX-585	Bispecific Ab (PD-1)	1	NCT05788484
**LILRB2**	ES009	mAb	1	NCT06007482
**LILRB2**	OR502	mAb	1/2	NCT06090266
**LILRB2; LILRB1**	NGM707	mAb	1/2	NCT04913337
**LILRB2; LILRB1**	IOS-1002	Fusion protein	1	NCT05763004
**LILRB4**	MK-0482	mAb	2	NCT03918278, NCT04165070, NCT04165096, NCT04165083, NCT04541108, NCT05038800
**LILRB4**	NGM831	mAb	1	NCT05215574
**LILRB4**	IO-202	mAb	1	NCT05309187

**PI3Kγ**	Eganelisib; IPI-549	Small molecule	2	NCT02637531, NCT03719326, NCT03980041, NCT03961698, NCT03795610
**PI3Kγ**	ZX-4081	Small molecule	1/2	NCT05118841
**PI3Kγ**	HS248	Small molecule	1	NCT05759234
**PI3Kγ; PI3Kδ**	Duvelisib; IPI-145	Small molecule	Appr.	NCT01476657, NCT01871675, NCT01882803, NCT02004522, NCT02049515, NCT02158091, NCT02204982, NCT02391545, NCT02292225, NCT02598570, NCT02711852, NCT02783625, NCT03372057, NCT03534323, NCT04038359, NCT03892044, NCT04707079, NCT03961672, NCT04193293, NCT04331119, NCT04209621, NCT04688658, NCT04803201, NCT05010005, NCT05057247, NCT04652960, NCT05065866, NCT04890236, NCT05044039, NCT05508659, NCT05675813, NCT05976997
**PI3Kγ; PI3Kδ; SIK3**	Tenalisib; RP6530	Small molecule	2	NCT02017613, NCT02567656, NCT03471351, NCT03711578, NCT03711604, NCT03770000, NCT04204057, NCT05021900
**PI3Kγ; PI3Kδ**	ZX-101A	Small molecule	1	NCT04504708, NCT05269940, NCT05258266
**Siglec-15**	NC318	mAb	2	NCT03665285, NCT04699123
**Siglec-15**	PYX-106	mAb	1	NCT05718557
**SIRPα**	CC-95251	mAb	1	NCT03783403, NCT05168202
**SIRPα**	BI 765063	mAb	1	NCT03990233, NCT04653142, NCT05068102, NCT05249426, NCT05446129
**SIRPα**	GS-0189	mAb	1	NCT04502706
**SIRPα**	BR105; lumistobart	mAb	1	NCT05351697
**SIRPα**	LM-101	mAb	1/2	NCT05615974
**SIRPα**	ADU-1805	mAb	1	NCT05856981
**SIRPα**	BYON4228	mAb	1	NCT05737628
**STAT3**	OPB-31121	Small molecule	1/2	NCT00511082, NCT00657176, NCT01029509, NCT00955812, NCT01406574
**STAT3**	Napabucasin; BBI608	Small molecule	3	NCT01775423, NCT01325441, NCT01776307, NCT01830621, NCT02024607, NCT02231723, NCT02178956, NCT02279719, NCT02347917, NCT02358395, NCT02315534, NCT02432326, NCT02352558, NCT02467361, NCT02641873, NCT02753127, NCT02851004, NCT02826161, NCT02993731, NCT03522649, NCT03647839, NCT03721744
**STAT3**	OPB-51602	Small molecule	1	NCT01184807, NCT01423903, NCT01344876, NCT02058017
**STAT3**	AZD9150; danvatirsen;	Anti-sense oligonucleotide	2	NCT01563302, NCT01839604, NCT02417753, NCT02499328, NCT02549651, NCT02546661, NCT02983578, NCT03334617, NCT03394144,

	IONIS-STAT3Rx			NCT03421353, NCT03527147, NCT03819465, NCT03794544, NCT05814666
**STAT3**	OPB-111077	Small molecule	2	NCT01711034, NCT01942083, NCT02250170, NCT03063944, NCT03158324, NCT03197714, NCT04049825
**STAT3**	TTI-101	Small molecule	1/2	NCT03195699, NCT05384119, NCT05440708, NCT06141031
**STAT3**	DSP-0337	Small molecule	1	NCT03416816
**STAT3**	WP1066	Small molecule	2	NCT01904123, NCT04334863, NCT05879250
**STAT3**	DCR-STAT3	Small interfering RNA	1	NCT06098651
**STAT6**	ExoASO-STAT6; CDK-004	Exosome-based delivery of anti-sense oligonucleotide	1	NCT05375604
**STING**	MIW815; ADU-S100	Small molecule	2	NCT02675439, NCT03172936, NCT03937141
**STING**	MK-1454; ulevostinag	Small molecule	2	NCT03010176, NCT04220866
**STING**	MK-2118	Small molecule	1	NCT03249792
**STING**	GSK3745417	Small molecule	1	NCT03843359, NCT05424380
**STING**	BMS-986301	Small molecule	1	NCT03956680
**STING**	IMSA101	Small molecule	2	NCT04020185, NCT05846646, NCT05846659, NCT06026254
**STING**	SB 11285	Small molecule	1	NCT04096638
**STING**	SYNB1891	Bacterial vector	1	NCT04167137
**STING**	E7766	Small molecule	1	NCT04144140
**STING**	TAK-676; dazostinag	Small molecule	1/2	NCT04420884, NCT06062602, NCT04879849
**STING**	exoSTING; CDK-002	Exosome-based delivery of STING agonist	1/2	NCT04592484
**STING**	SNX281	Small molecule	1	NCT04609579
**STING**	HG381	Small molecule	1	NCT04998422
**STING**	TAK-500	Antibody–drug conjugate (CCR2)	1/2	NCT05070247
**STING**	KL340399	Small molecule	1	NCT05387928, NCT05549804
**STING**	BI 1387446	Small molecule	1	NCT04147234
**STING**	BI 1703880	Small molecule	1	NCT05471856
**STING**	XMT-2056	Antibody–drug conjugate (HER2)	1	NCT05514717

**STING**	CRD3874	Small molecule	1	NCT06021626
**STING**	ONM501	STING-agonist in polymeric micelles	1	NCT06022029
**TLR2; TLR4**	OM-174; CRX-527	Synthetic lipid A analogue	1	NCT01800812
**TLR3**	Hiltonol; poly-ICLC	Nucleic-acid based	2	NCT00052715, NCT00058123, NCT00553683, NCT00880867, NCT01188096, NCT01984892, NCT02061449, NCT02643303, NCT03162562, NCT02834052, NCT03721679, NCT04116320, NCT05281926, NCT04544007, NCT06064279
**TLR3**	Rintatolimod; ampligen	Nucleic-acid based	2	NCT01545141, NCT03403634, NCT03599453, NCT03734692, NCT03899987, NCT04081389, NCT04379518, NCT05927142, NCT05494697, NCT05756166
**TLR3**	BO-112	Nucleic-acid based	2	NCT02828098, NCT04508140, NCT04420975, NCT04570332, NCT04777708, NCT05265650
**TLR4**	G100; GLA-SE	Synthetic lipid A derivative	2	NCT02035657, NCT02180698, NCT02406781, NCT02501473
**TLR4**	GSK1795091	Synthetic lipid A analogue	1	NCT03447314
**TLR5**	entolimod; CBLB502	Recombinant protein	2	NCT01527136, NCT02715882
**TLR5**	mobilan; M-VM3	Viral vector for TLR5 and entolimod	1/2	NCT02654938, NCT02844699
**TLR7**	Imiquimod; aldara; TMX-101; UGN-201	Small molecule	Appr.	NCT00031759, NCT00189241, NCT00189306, NCT00204555, NCT00066872, NCT00189280, NCT00129519, NCT00079300, NCT00314756, NCT00707174, NCT00273910, NCT00453050, NCT00384124, NCT00504023, NCT00581425, NCT00785122, NCT00941811, NCT00803907, NCT00821964, NCT01212549, NCT00865644, NCT00899574, NCT00941252, NCT01161888, NCT01088737, NCT01264731, NCT01421017, NCT01720407, NCT01731652, NCT01283763, NCT01861535, NCT02135419, NCT02329171, NCT02130323, NCT02394132, NCT02059499, NCT02242929, NCT02669459, NCT02917746, NCT00463359, NCT03206138, NCT03180684, NCT03276832, NCT03233412, NCT03116659, NCT04279535, NCT03370406, NCT04859361, NCT03872947, NCT03534947, NCT03196180, NCT03982004, NCT05055050, NCT04883645, NCT05375903
**TLR7**	PF-4878691; 852-A	Small molecule	2	NCT00095160, NCT00091689, NCT00189332, NCT00276159, NCT00319748
**TLR7**	LHC-165	Small molecule	1	NCT03301896
**TLR7**	DSP-0509; guretolimod	Small molecule	1/2	NCT03416335

**TLR7**	NJH395	Antibody–drug conjugate (HER2)	1	NCT03696771
**TLR7**	BNT411	Small molecule	1/2	NCT04101357
**TLR7**	TQ-A3334	Small molecule	1/2	NCT04273815
**TLR7**	RO7119929	Small molecule prodrug	1	NCT04338685
**TLR7**	SHR2150	Small molecule	1/2	NCT04588324
**TLR7**	MBS8(1V270)	Micellar TLR-agonist	1	NCT04855435
**TLR7**	CAN1012	Small molecule	1	NCT04987112, NCT05580991
**TLR7; TLR8**	Resiquimod; R848; STM-416	Small molecule	1/2	NCT01676831, NCT01808950, NCT05710848
**TLR7; TLR8**	telratolimod; MEDI9197	Small molecule	1	NCT02556463
**TLR7; TLR8**	BDB001; EIK1001	Small molecule	2	NCT03486301, NCT04196530, NCT03915678, NCT04840394, NCT04819373, NCT06246110
**TLR7; TLR8**	NKTR-262	Small molecule	1/2	NCT03435640
**TLR7; TLR8**	BDC-1001	Antibody–drug conjugate (HER2)	2	NCT04278144, NCT05954143
**TLR7; TLR8**	TransCon	Hydrogel with resiquimod	2	NCT04799054, NCT05081609, NCT05980598
**TLR7; TLR8**	BDB018	Small molecule	1	NCT04840394
**TLR8**	motolimod; VTX-2337	Small molecule	2	NCT00688415, NCT01294293, NCT01334177, NCT01289210, NCT01666444, NCT01836029, NCT02124850, NCT02431559, NCT02650635, NCT03906526, NCT04272333
**TLR8**	DN-1508052	Small molecule	1	NCT03934359
**TLR8**	SBT6050	Antibody–drug conjugate (HER2)	1/2	NCT04460456, NCT05091528
**TLR9**	PF-03512676; CpG 7909	Nucleic-acid based	2	NCT00031278, NCT00043407, NCT00040950, NCT00043394, NCT00043368, NCT00043420, NCT00070629, NCT00369291, NCT00070642, NCT00233506, NCT00185965, NCT00438880, NCT00880581, NCT00824733
**TLR9**	EMD 1201081; IMO-2055	Nucleic-acid based	2	NCT00729053, NCT00633529, NCT00719199, NCT01040832, NCT01360827
**TLR9**	GNKG168	Nucleic-acid based	1	NCT01035216, NCT01743807
**TLR9**	SD-101	Nucleic-acid based	2	NCT01042379, NCT01745354, NCT02254772, NCT02266147, NCT02521870, NCT02731742, NCT02927964, NCT03007732, NCT03322384,

				NCT03410901, NCT03831295, NCT04050085, NCT04935229, NCT05220722, NCT05607953
**TLR9**	MGN1703; lefitolimod	Nucleic-acid based	3	NCT01208194, NCT02200081, NCT02077868, NCT02668770
**TLR9**	CMP-001; vidutolimod	Virus-like vector for a TLR9 agonist	<u>2</u>/3	NCT02554812, NCT02680184, NCT03084640, NCT03438318, NCT03618641, NCT03507699, NCT03983668, NCT04401995, NCT04633278, NCT04695977, NCT04698187, NCT04807192, NCT04387071, NCT04708418, NCT04916002, NCT05445609
**TLR9**	tilsotolimod; IMO-2125	Nucleic-acid based	3	NCT02644967, NCT03052205, NCT03445533, NCT03865082, NCT04126876, NCT04196283, NCT04270864
**TLR9**	DV281	Nucleic-acid based	1	NCT03326752
**TLR9**	cavrotolimod	Nucleic-acid based	1/2	NCT03684785
**TREM1**	PY159	mAb	1	NCT04682431
**VISTA**	CI-8993; Onvatilimab; JNJ-61610588	mAb	1	NCT02671955, NCT04475523
**VISTA; PD-L1**	CA-170	Small molecule	1	NCT02812875
**VISTA**	W0180	mAb	1	NCT04564417
**VISTA**	HMBD-002	mAb	1	NCT05082610
**VISTA**	KVA-12123	mAb	<u>1</u>/2	NCT05708950
**VISTA**	SNS-101	mAb	<u>1</u>/2	NCT05864144
**VISTA**	PMC-309	mAb	1	NCT05957081

*Therapeutics for the same target and their trial IDs were ordered based on trial start date. Former drug names were omitted, if they did not yield clinicaltrials.gov search results. For TLR agonists, trials investigating the TLR agonist as an adjuvant to cancer vaccine or dendritic cell therapy were excluded.
**Highest trial phase. For phase 1/2 and phase 2/3 trial designs, underlined numbers indicate the phase reported in company pipeline in January 2024.
Ab, antibody; Appr., approved; FAP, fibroblast activation protein alpha; mAb, monoclonal antibody; NA, not available.
